# Antimicrobial activity of *Spirulina platensis* extract on total mesophilic and psychrophilic bacteria of fresh tilapia fillet

**DOI:** 10.1038/s41598-023-40260-z

**Published:** 2023-08-11

**Authors:** Wubshet Asnake Metekia, Beyza Hatice Ulusoy

**Affiliations:** 1 Ethiopian Ministry of Agriculture, Food and Nutrition Office, Food Safety and Quality Desk, P. O. Box. 62347, Addis Ababa, Ethiopia; 2 Food Hygiene and Technology Department, Faculty of Veterinary Medicine, Near East University, 99138 Nicosia, Cyprus

**Keywords:** Antimicrobials, Bacteria

## Abstract

*Spirulina platensis* has a wide range of activities, notably antibacterial property against food pathogens. This study investigates the antibacterial activity of *S. platensis* extract on Total Mesophilic and Psychrophilic Aerobic Bacteria. The results were compared using statistical analysis and the predicted model values using artificial intelligence-based models such as artificial neural network (ANN) and adaptive neuro fuzzy inference system (ANFIS) Models. The extraction of spirulina was done by using the freeze–thaw method with a concentration of 0.5, 1 and 5% w/v. Before the application of the extract, initial microbial load of fillets was analyzed the and the results were used as control. After application analysis was performed at 1, 24 and 48 h of storage at 4 °C. Based on the statistical analysis result the *S. platensis* extracts’ antimicrobial activity over TMAB of fresh tilapia fish fillets at 1, 24 and 48 h was using EA from 2.5 log10 CFU/g during the control stage to 1.8, 1.1 and 0.7 log10 CFU/g respectively whereas EB and EC was from 2.1 and 2.2 log10 CFU/g at control to 1.5, 0.8, 0.5 log10 CFU/g and 1.23, 0.6 and 0.32 log10 CFU/g respectively at the specified hour interval. Similarly, the three extracts over TPAB were from 2.8 log10 CFU/g at control time to 2.1, 1.5 and 0.9 in EA, while using EB reduces from 2.8 log10 CFU/g to 1.9, 1.3 and 0.8 log10 CFU/g at 1, 24 and 48 h respectively. Although EC presented the reduction from 1.9 log10 CFU/g to 1.4, 1 and 0.5 log10 CFU/g. This was supported by ANN and ANFIS models prediction.

## Introduction

Seafood products are highly perishable and rapidly changed its quality after harvest, this is because of temperature that allows the growing of foodborne pathogens and spoilage microorganisms and reduces the shelf-life of the food^1^. Researchers, the food business, consumers, and health professionals have all been paying close attention to various seafood preserving methods in recent years. Various natural preservatives from various sources, such as chitosan from animal origins, essential oils, and plant extracts from a plant source, lactic acid bacteria, and bacteriocins from microbiological sources, and organic acid from various sources, have all been thoroughly researched and demonstrate great promise for use in seafood systems^1^.

Supporting studies also showed that the natural citric acid and lactic acid together with ice inhibits bacterial growth and enhances the quality of fresh fish fillets of hake and megrim species and those natural preservatives were considered as a good strategy to increase the market value and deliver quality fresh fish fillets product to the consumer^2^. The mix of nisin and grape seed extract serve as an antimicrobial agent in the control and inhibition of Listeria monocytogenes in ready to eat shrimp fillets^3^.

Spirulina is another important natural preservatives and antimicrobial agent for food pathogenic bacteria and fungi including drug resistant microorganisms. Spirulina have been known as a food supplement, natural colorant and good bio-active secondary metabolites source including phenolic compounds^3,4^.

Globally Spirulina is identified and taken as for its big nutritional values, major ingredients in the development of novel functional food, high phycocyanin content, excellent health remedy to many disorders^5–7^, helps in non-communicable disease^8,9^, for development of functional foods and antioxidant agent with long shelf life of the product^10,11^.

Spoilage of fishery products occur immediately when it out from the natural water as a result of the enzymes’ activities, oxidation and because of pathogenic and spoilage microorganisms^12,13^. Worldwide the food spoilage is very high, like 25% of the world’s food supply and 30% of the fishery products are spoiled and discarded because of undesirable microorganisms^14^. So that food preservation turns into an important matter in the food industry to keep its quality, freshness and increases the shelf life of the product and reduce public health risks. In line with this, those highly perishable fishery products were traditional preserved using different methods including salting, sun drying, smoking, fermentation, canning, cooling, freezing and addition of chemicals^14^. This is supported by Tsironi et al.^13^ and recently the introduction of new fish processing novel technologies is also a good solution including methods like high hydrostatic pressure, osmotic dehydration, high-intensity pulsed light and modified atmospheric packaging and other combined methods. But this also have some limitations, like some pathogenic microorganisms resist after the processing for example psychrotolerant lactobacilli and also some processing techniques were affected the nutritional and sensory properties of the fishery product^13^.

Still researchers are searching and focusing on different natural extracts from different sources including spirulina algae. Together with this many recent studies showed that Spirulina algae extracted compounds displayed good results in controlling food pathogenic microorganisms and serve as a preservatory and an antimicrobial activator in fish and fish products^4,15–17^.

On the other hand, Artificial Intelligence (AI) based models are currently being utilized in many production systems to evaluate, simulate, and forecast the process and interaction of numerous input and output factors. Metekia et al.^17^ studied the effect of spirulina growth mediums on phenolic compounds using the Artificial Intelligence based models; ANFIS and ANN together with SWLR. And the researchers find out, total phenolic compounds had high positive correlation with growth mediums and the ANFIS and SWLR gives excellent prediction than the ANN model. Thus, this current study was planned to evaluate the antimicrobial efficiency of *S. platensis* extract on fresh tilapia fish fillets by using artificial intelligence-based models.

## Material and methods

### Extraction of spirulina

**Figure 1:**
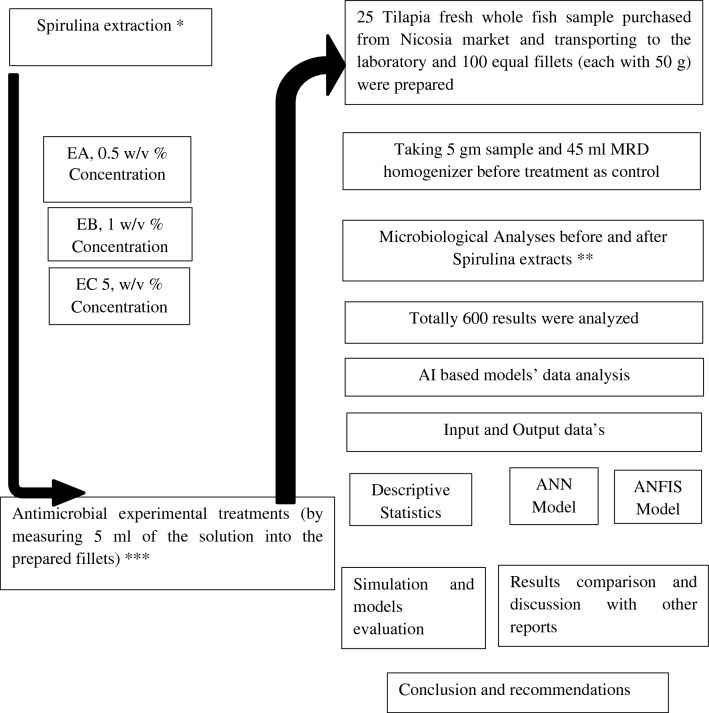
Flow chart for the experimental study of antimicrobial activity of *S. platensis* extracts on fresh tilapia fish fillets.

The fresh blue-green algae *S. platensis* was brought from Çukurova University, Adana, Turkey. The ready biomass of *Spirulina platensis* was stored in -18 °C in freezer for the next step of extraction in Food Hygiene and Technology Department, Faculty of Veterinary Medicine in Near East University within hygienic packages. The extraction of Spirulina was done using the freeze–thaw method based on Tan et al.^18^ with some modifications on the concentrations 0.5, 1 and 5 g of freeze Spirulina were weighted with 100 ml of sterilized distilled water for each group of solutions and assigned as Extract A (EA), Extract B (EB) and Extract C (EC). A maceration of the cells with the degradation of proteins and the extraction of polysaccharides will perform antimicrobial role over pathogenic bacteria. The Hu angle was used for blue color valuation of the Spirulina extract^19,20^. Keeping the fresh Spirulina biomass in the freezer at − 18 °C for 2 h; is the freezing stage (first step). At the second step 0.5, 1 and 5 g of Spirulina biomass were weighted from the freeze stock and were put in to the labeled and sterilized bottles and then were measured and added 100 ml distilled water. The bottles were then slightly mixed for a few minutes, kept in water bath at 25 °C and covered with aluminum sheet to create dark environment for the extraction process for 24 h. After 24 h the supernatant was separated and stored at + 4 °C. Please look Fig. 1. that show the flow chart for the experimental study of antimicrobial activity of *S. platensis* extracts on fresh tilapia fish fillets.

### Preparing fish samples and experimental design

Twenty-five fresh whole Nile tilapia fishes (*Oreochromis niloticus*) were purchased from Nicosia fish market and dissected and fileted, each with 50 g weight. The prepared fish fillets were placed in sterile plates where the extracts were also applied on the samples. Before the application of the extract from each fresh fillet we took and analyzed the initial microbiota count and taken as control. Enumeration. Each samples of fillets were treated by Spirulina extracts coded as EA, EB and EC concentrations with 0.5, 1 and 5 w/v concentrations, respectively. Throughout the experiment aseptic conditions were provided and alcohol/flame were used to sterilize all materials that contact with samples. The microbial analysis was compared; total viable count on plate of Total Mesophilic Aerobic Bacteria (TMAB) and Total Psychrophile Aerobe bacteria (TPAB) before and after Spirulina platensis extract application treatments. The fish fillets were stored at refrigerator at 4 °C and after 1, 24 and 48 h microbiological analysis were performed to evaluate the antimicrobial activity of the extracts.

### Microbiological analysis of TMAB and TPAB

5 g of the tilapia fish fillets samples were weighed before treatment and after treatment from each sample into a sterile glass jar together with 45 mL Maximum Recovery Diluent (MRD) solutions homogenizer and serial dilutions of 1:10 were done^21^. Total Mesophilic Aerobic Bacteria (TMAB) and Total Psychrophile Aerobe bacteria (TPAB) were enumerated on Plate Count Agar (PCA) after incubation at 37 °C for 48 h TMAB and at 10 °C for 7 days TPAB enumeration were performed. Results were expressed as log10 CFU/g^22^.

### Evaluation of the results by artificial intelligence

Artificial Neural Network (ANN), Adaptive-Neuro Fuzzy Inference System (ANFIS) and descriptive statistics were used to analyze and compare the activities of Spirulina algae extracts antibacterial action on TMAB and TPAB of fresh tilapia fish fillets. The Adaptive-Neuro Fuzzy Inference System (ANFIS) is used in artificial intelligence to estimate various types of issues. The ANFIS is composed of two fundamental layers: feed forward networks and adaptive multi-layer networks. Again, feed-forward networks use fuzzy Takagi–Sugeno type instructions to include input–output variables. The fundamental components of the layout in the fuzzy data-base system are the fuzzyer and defuzzifier. The membership functions used in fuzzy logic include converting input values into fuzzy data. Nodes that serve as membership functions aid in modeling the link between inputs and outputs. Therefore, node work as a connection function, permits the modelling of the relation between the input and outputs scheme. There are many distinct types of connection functions, such as triangular, sigmoid, Gaussian, and trapezoidal^17,23^. There are two essential considerations in the technique that should be taken into account both from the input and output arrangements, firstly the two variables of the FIS 'x' and 'y' inputs data and one output 'f', a first-order Sugeno fuzzy and as a rule it follows the following formula.

1 $$ {\text{Method}}\;{1}:\;{\text{if}}\;\mu \left( {\text{x}} \right)\;{\text{is}}\;{\text{A}}_{1} \,{\text{and}}\;\mu \left( {\text{y}} \right)\;{\text{B}}_{1} \;{\text{then}}\;{\text{f}}_{1} = \;{\text{p}}_{1} {\text{x}} + {\text{q}}_{1} {\text{y}} + {\text{r}}_{1} $$

2 $$ {\text{Method}}\;{2}:\,{\text{if}}\;\mu \left( {\text{x}} \right)\;{\text{is }}\;{\text{A}}_{2} \;{\text{and}}\;\mu \left( {\text{y}} \right)\;{\text{is }}\;{\text{B}}_{2} \;{\text{then}}\;{\text{f}}_{2} = \;{\text{p}}_{2} {\text{x}} + {\text{q}}_{2} {\text{y}} + {\text{r}}_{2} $$

where ${\mathrm{A}}_{1}$,${\mathrm{B}}_{1},{\mathrm{A}}_{2},{\mathrm{B}}_{2}$ constraints are membership functions for x and y, and inputs ${p}_{1},{q}_{1},{r}_{1,}{p}_{2},{q}_{2},{r}_{2,}$ are outlet function parameters. The building and design of ANFIS follows a five-layer neural network arrangement^17^.

One of the most used examples of an ANN that aids in the operation and solution of non-linear systems is the multilayer perceptron (MLP) neural network. Several academics believe that this estimator is commonly recognized when compared to the other types of ANNs. The Multilayer Perceptron (MLP) neural network is constructed similarly to other conventional ANN models, using input and output layers, among them is a hidden input layer^23^.

As a learning algorithm, the Levenberg–Marquardt algorithm is commonly used to fix and minimize the variation among measured and projected values. Until the intended outcomes become apparent, the training procedures are repeatedly performed. The MLP comprises an input, one or more hidden layers, and output layers, just like a conventional ANN^24^. Together with this the considerations in the inputs data set were the Spirulina extracts concentrations EA, EB and EC (0.5, 1 and 5) w/v and the initial microbial load as a control microorganism (log10 CFU/g) and the output data consideration was the result obtained by Spirulina antimicrobial activity or microbial reduction using Spirulina extracts at 1, 24 and 48 h (log10 CFU/g), the flow chart of the study was displayed above in Fig. 1.

3 $$ y_{i} = \mathop \sum \limits_{j = 1}^{N} w_{ji} x_{j} + w_{i0} $$

where N is the total number of nodes in the top layer of the node, i; w_ji_ is the weight between the nodes i and j in the upper layer; x_j_ defines the output derived from node j; w_i0_ is the bias in node i, and y_i_ describes the input signal of node i which crosses via the transfer function.

The data was from the laboratory experimental study findings at Food Hygiene and Technology Department, Veterinary Medicine Faculty of Near East University, Nicosia, Cyprus. In this analysis the initial microbial load before treatment as the control microbial load before treatment (CFU log10/g) and the results from each treated samples were taken as an input variables. Total bacterial load reduction after treatment at the specified time interval i.e., 1, 24 and 48 h (CFU log10/g) were taken as output variables in the analysis and modeling of this study.

### Evaluation criteria for data—driven models

The performance accuracy of any type of data-driven study is usually determined by comparing projected values to measured values. To estimate the models, the determination coefficient (DC) as a goodness of fit, correlation coefficient (CC), and two statistical errors, root mean-squared error (RMSE) and mean-squared error (MSE), were used^17^.

4 $$ DC = 1 - \frac{{\mathop \sum \nolimits_{j = 1}^{N} \left[ {\left( Y \right)_{obs,j} - \left( Y \right)_{com,j} } \right]^{2} }}{{\mathop \sum \nolimits_{j = 1}^{N} \left[ {\left( Y \right)_{obs,j} - \overline{\left( Y \right)}_{obs,j} } \right]^{2} }} $$

5 $$ CC = \frac{{\mathop \sum \nolimits_{i = 1}^{N} \left( {Y_{obs} - \overline{Y}_{obs} } \right)\left( {Y_{com} - \overline{Y}_{com} } \right)}}{{\sqrt {\mathop \sum \nolimits_{i = 1}^{N} \left( {Y_{obs} - \overline{Y}_{obs} } \right)^{2} } \mathop \sum \nolimits_{i = 1}^{N} \left( {Y_{com} - \overline{Y}_{com} } \right)^{2} }} $$

6 $$ RMSE = \sqrt {\frac{{\mathop \sum \nolimits_{i = 1}^{N} \left( {Y_{obsi} - Y_{comi} } \right)^{2} }}{N}} $$

7 $$ {\text{MSE}} = \frac{1}{N}{ }\mathop \sum \limits_{i = 1}^{N} (Y_{obsi} - Y_{comi} )^{2} $$

where N, ${Y}_{obsi}$, $\overline{Y }$ and ${Y}_{comi}$ are data number, observed data, average value of the observed data and computed values, respectively.

### Data set description and validation of the models

The basic goal of a data-driven scheme is to prepare data for models based on functional needles for a specific value set in order to provide accurate and consistent predictions of unknown data sets. Overfitting values or acceptable working activities are usually ignored in this method. As a result, different methods of verification, cross-validation, and proof were used in the endorsement stage, such as k-fold cross-validation, holdout; leave one out, and so on. The most fundamental benefit of the k fold proof tool is that the verification and working sets are self-determining in every single point. As previously stated, the data is further divided into two groups: 75 percent for training and 25 percent for testing, with k-fold cross-validation also being important. Another significant point to mention about this process is the data validation methods we used^23^. The data set consists of 25 occurrences for each of the variables.

### Patient and public involvement

The study was conducted on fresh fish fillets from a food hygiene and safety perspective, hence there was no patient or public involvement in this study.

## Results and discussion

*Spirulina platensis* shows antimicrobial activity against many pathogenic bacteria and fungi^25^ by its functional bioactive ingredients including phenolic phytochemicals^26^. Antimicrobial compounds found in cyanobacterial exudates include polyphenols, fatty acids, glycolipids, terpenoids, alkaloids and especially the C-phycocyanin. In small concentration, phycocyanin effectively acts against many pathogens^27^. In this experimental study finding the three different *Spirulina platensis* extracts concentrations, EA, EB and EC antimicrobial activity on total mesophilic aerobic bacteria (TMAB) were different based on statistical analysis the concentration of the extracts like using EA the reduction was from 2.5 log10 CFU/g during the control stage to 1.8, 1.1 and 0.7 log10 CFU/g at 1, 24 and 48 h respectively whereas using EB it reduces from 2.1 log10 CFU/g to 1.5, 0.8 and 0.5 log10 CFU/g at 1, 24 and 48 h respectively. Although EC presented the reduction from 2.2 log10 CFU/g at control time to 1.23, 0.6 and 0.32 log10 CFU/g at the specified hour interval respectively, please look the details at Table 1.

**Table 1:** *S. platensis* extracts antimicrobial activity on TMAB descriptive statistic summery in log10 CFU/g.

	*EA*				*EB*				*EC*			
	C	1 h	24 h	48 h	C	1 h	24 h	48 h	C	1 h	24 h	48 h
Mean	2.5	1.8	1.1	0.7	2.1	1.5	0.8	0.4	2.2	1.2	0.6	0.3
Median	2.7	1.7	1.2	0.6	1.5	1.2	0.7	0.4	1.8	1.0	0.3	0.2
Mode	3.0	1.6	1.2	1.0	1.1	1.0	0.9	0.4	1.6	0.3	0.2	0.0
Standard Deviation	0.7	0.6	0.6	0.5	1.3	0.9	0.6	0.4	0.9	0.9	0.5	0.3
Kurtosis	0.2	3.4	1.5	1.0	7.5	7.6	15.7	9.7	−0.7	−0.5	0.5	−0.6
Range	2.9	2.7	2.8	1.8	5.9	4.2	3.3	1.9	3.3	2.9	1.8	0.9
Minimum	0.9	0.9	0.0	0.0	0.9	0.7	0.1	0.0	0.8	0.2	0.0	0.0
Maximum	3.8	3.6	2.8	1.8	6.8	4.9	3.3	1.9	4.1	3.1	1.8	0.9

**Figure 2:**
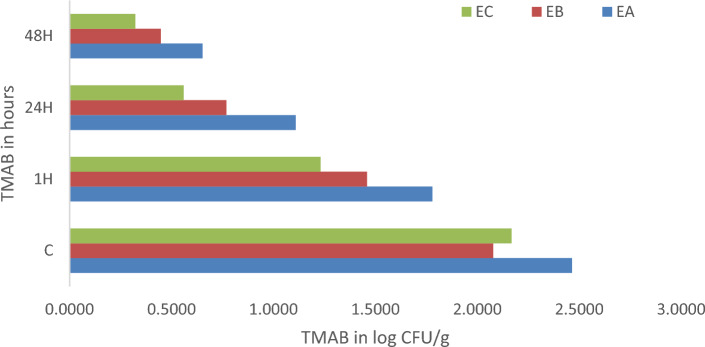
Average Spirulina extracts antimicrobial activity on TMAB using bar graph at different time duration.

**Figure 3:**
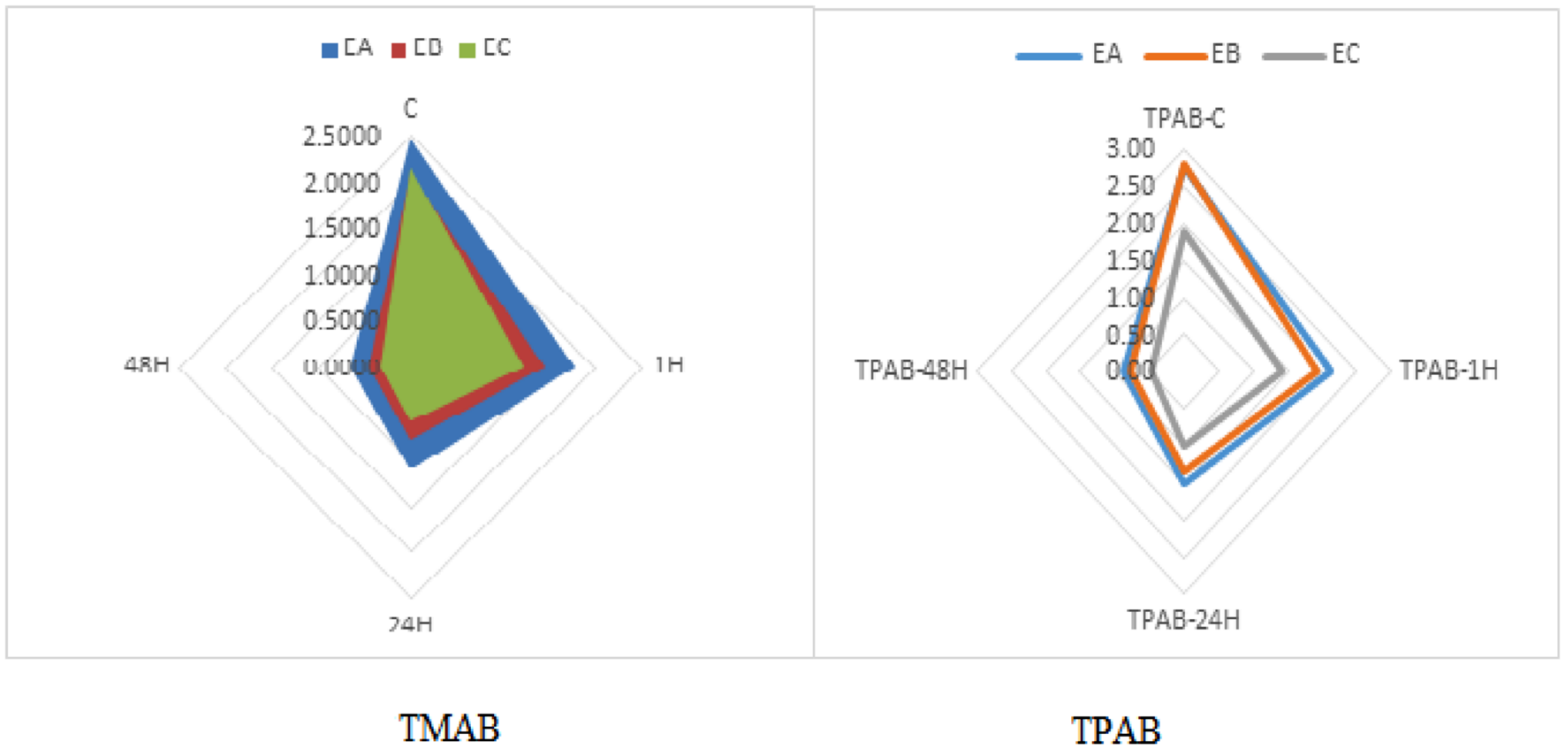
Average Spirulina extracts antimicrobial activity on TMAB and TPAB using Radar chart graph at different time duration.

In the one-way ANOVA analysis of the Spirulina extracts antimicrobial activity on TMAB with F value of 77.05; *P* value: 7.33E−37, which is around to 0 and F critical: 2.64. As the F-statistic is greater than F-critical value then the test is significant. And *P* ≤ 0.05 then we reject the null hypothesis and we accept the alternate hypothesis i.e., *Spirulina platensis* extracts had antimicrobial activity role over spoilage microorganisms of fresh fish fillets; which is the Spirulina extracts had significant antimicrobial activity as compare to the control over TMAB. The *S. platensis* extracts antimicrobial activity over the TMAB was also supported by the line-graph, bar-graph and radar chart as shown in Figs. 2 and 3 below, EC had better antimicrobial role than EB and EA on TMAB control and inhibition. Whereas, the ANN model predicted the activity of all the three *S. platensis* extracted EA, EB and EC antimicrobial activity on TMAB were analyzed and simulated at the specified time of 1, 24 and 48 h.

Together with this the overall average ANN modeling of the Spirulina extracts antimicrobial activity on TMAB was 1.71, 1.13, 0.71 log10 CFU/g using EA, and 1.68, 0.91, 0.52 log10 CFU/g using EB and 1.23, 0.55, 0.3 log10 CFU/g using EC respectively. This is also supported by the regression analysis of the three extracts at training, validation and testing stages of the ANN model, like EA with R = 0.9228, 0.8408, 0.9419 respectively. While EB over TMAB had good correlation with R = 0.90974 at the training stage, R = 0.8904 at validation stage, and R = 0.9244 at testing stage. Whereas EC also had a good correlation with R = 0.8712 at the training stage, R = 0.9409 at validation stage, and R = 0.9586 at testing stage. Also, the ANFIS model anticipated the antimicrobial role of *S. platensis* extracts (EA, EB and EC) activity over TMAB was 1.78, 1.20, 0.74 log10 CFU/g using EA, and 1.55, 0.83, 0.51 log10 CFU/g using EB and 1.40, 0.66, 0.39 log10 CFU/g using EC at 1, 24 and 48 h respectively. In both the models the MSE and RMSE was between 0 and 0.5. Another study also supports this finding with both models i.e., ANN and ANFIS produced best predictions with the experimental findings^28^.

**Figure 4:**
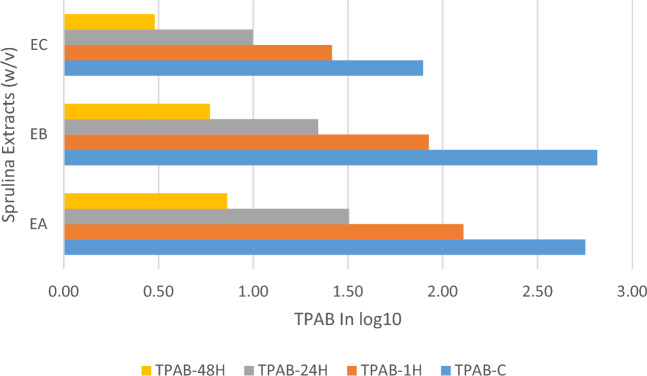
The Spirulina extracts antimicrobial activity on TPAB reduction using bar chart.

Whereas the *S. platensis* extracts, EA, EB and EC mean antimicrobial activity against TPAB had statistically analyzed and the antimicrobial activity was from 2.8 log10 CFU/g at control time to 2.1, 1.5 and 0.9 in EA, while using EB reduces from 2.8 log10 CFU/g to 1.9, 1.3 and 0.8 log10 CFU/g at 1, 24 and 48 h respectively. Although EC presented the reduction from 1.9 log10 CFU/g to 1.4, 1 and 0.5 log10 CFU/g at the specified hour interval respectively, please look Table 2. In the one-way ANOVA analysis of the Spirulina extracts antimicrobial activity had F-value: 52.90, *P* value: 2.09E−27 which is around 0 and F-critical 2.65. The test is significant i.e., the *S. platensis* extract showed good antimicrobial result over TPAB, EC had superior antimicrobial role than EB and EA against TPAB as presented in Figs. 3 and 4 below, please look it. In other study the Spirulina algae extract phenolic compound controls gram positive and negative bacteria^4,29^.

**Table 2:** *S. platensis* extracts antimicrobial activity on TPAB descriptive statistic summery and results are in log10 CFU/g.

	TPAB–EA				TPAB–EB				TPABA–EC			
	C	1 h	24 h	48 h	C	1 h	24 h	48 h	C	1 h	24 h	48 h
Mean	2.8	2.1	1.5	0.9	2.8	1.9	1.3	0.8	1.9	1.4	1.0	0.5
Standard Error	0.2	0.2	0.2	0.1	0.2	0.2	0.2	0.1	0.2	0.2	0.2	0.1
Median	2.3	1.8	1.3	0.8	2.7	1.6	1.0	0.7	1.4	0.9	0.6	0.5
Mode	2.0	1.8	NA	0.0	2.3	1.4	1.7	0.0	1.2	0.7	2.1	0.0
Standard Deviation	1.1	0.8	0.7	0.6	1.1	1.2	0.9	0.6	1.0	0.9	0.8	0.4
Kurtosis	− 0.1	− 0.6	− 0.9	− 1.0	1.8	2.5	1.6	0.1	− 1.1	− 1.0	− 1.4	− 1.3
Skewness	1.1	0.7	0.4	− 0.1	1.3	1.4	1.4	0.7	0.6	0.7	0.5	0.2
Range	3.7	2.9	2.5	1.8	4.3	5.1	3.6	2.1	3.1	2.8	2.4	1.2
Minimum	1.5	0.9	0.3	0.0	1.4	0.1	0.2	0.0	0.7	0.4	0.1	0.0
Maximum	5.1	3.8	2.9	1.8	5.7	5.1	3.8	2.1	3.8	3.1	2.4	1.2

The ANN modeling of the Spirulina extracts antimicrobial activity over TPAB was 2.27, 1.72, 0.95 log10 CFU/g using EA, and 2.03, 1.45, 0.86 log10 CFU/g using EB and 1.64, 1.20, 0.58 log10 CFU/g using EC at 1, 24 and 48 h respectively. This is also supported by the regression analysis at training, validation and testing stage with EA of R = 0.9359, 0.8962, 0.9141 respectively. While EB over TPAB had a correlation of R = 0.8866, 0.9248, 0.9579 at the training, validation and testing stages respectively. Although EC performs best result and good correlation in the training, validation and testing stages with R = 0.9604, 0.9535 and 0.9763 respectively. Whereas MSE and RMSE was between 0 and 0.2. Also, the ANFIS model projected the antimicrobial role of *S. platensis* extracts (EA, EB and EC) over TPAB with an overall average modeling of 2.53, 1.85, 1.02 log10 CFU/g by EA, and 1.90, 1.36, 0.76 log10 CFU/g using EB and 1.71, 1.20, 0.60 log10 CFU/g using EC at 1, 24 and 48 h respectively. Another supportive study was reported by Yolmeh et al.^28^, Supportive study were reported that 1% v/w microalgae concentration extracts (*Spirulina platensis* and *Chlorella vulgaris*) controls bacterial growth and extends the shelf life of the vacuum packed and chilled stored sardine for three days and *Spirulina platensis* showed superior antibacterial role than *Chlorella vulgaris*^29^. Whereas in another trail the minimum inhibiter concentration of Spirulina antimicrobial concentration was 8 and 16 mg/ml for *E. coli* and *S. aureus* bacteria respectively^30^.

In another study the Spirulina algae extract using methanol, acetone and hexane and selenium nano-particles developed by *Bacillus subtilis* experiment exhibited that, Spirulina methanol extract produced high total phenolic compound amount with good antimicrobial and antioxidant role than other extracts. And its antibacterial were active both gram positive and negative bacteria and as well over fungal organisms as antifungal activity including *Candida* and *Aspergillus* *species*^15^. Whereas another study showed phenolic compounds from fermented race bran and Spirulina species LEB-18 inhibited fungal growth by 39.8 and 20.2% respectively, and ochratoxin A by 40.2 and 29% respectively (Christ-Ribeiro et al.^16^). One more study provided that ANFIS models presented superior result as compare with other models like ANN^31^.

Generally, the study showed that the *Spirulina platensis* extracts EA, EB and EC had good active antimicrobial activity efficiency (count reduction) on both the targeted fresh fish fillet microorganisms TMAB and TPAB. This were supported by the detail analysis of the descriptive statistics, ANN and ANFIS model prediction. Additionally, in all the experimental activity there were significant difference between the initial bacterial load (the control) and treatments.

Overall, *S. platensis* extract EC gives superior result than EB and EA. As well as EB also were better than EA in this study. So, when the concentration of the Spirulina increases from 0.5 to 1 and 5% w/v concentration then its antimicrobial activity also increased and had better on the control on the fresh fish fillets spoilage microorganisms. Both the ANN and ANFIS models give good prediction result on the role of *S. platensis* extracts with different concentration (0.5, 1 and 5% w/v) antimicrobial activity on TMAB and TPAB. Hence, spirulina algae could be an emerged sustainable natural bioactive compound technology sourced from food to another seafood, fish filles preservatives for future use. As a recommendation other researcher better to focus on *S, platensis* bio-active compounds antimicrobial activity mechanism and its chemical compounds which are responsible for the activity. Also, a repeated freezing/thawing procedure with the extraction of phycobiliproteins with different concentration should be experimented.

### Supplementary Information

Supplementary Information.

## Data Availability

All data generated and/or analysed during this study are included in this published article and its supplementary information files.

## Abbreviations

*A. flavus*: *Aspergillus flavus*
AI: Artificial intelligence
ANFIS: Adaptive neuro fuzzy inference system
ANN: Artificial neural network
*A. platensis*: *Arthrospira platensis*
C: Control
*E. coli*: *Escherichia coli*
EA: Extract A
EB: Extract B
EC: Extract C
h or hs: Hour or hours
Log10 CFU/g: Log10 colony forming unit per gram
MLP: Multi-layer perceptron
MRD: Maximum recovery diluent
MSE: Mean square error
RBF: Radial basis function
RMSE: Root mean square error
RSM: Response surface methodology
*S. aureus*: *Staphylococcus aureus*
SP or *S. platensis*: *Spirulina platensis*
SWLR: Step-wise-linear regression
TMAB: Total mesophilic aerobic bacteria
TPAB1: Total psychrophile aerobe bacteria (TPAB)
