# Supplementary material for: Antimicrobial activity of Spirulina platensis extract on total mesophilic and psychrophilic bacteria of fresh tilapia fillet
Source: Sci Rep. 2023 Aug 11;13:13081. doi: 10.1038/s41598-023-40260-z (PMC10421913; doi:10.1038/s41598-023-40260-z)
Supplement: Supplementary file 1 — Supplementary Information. [file 41598_2023_40260_MOESM1_ESM.docx]

**Tables**

### **Table S1:** *S. platensis* extracts antimicrobial activity on TMAB modeling using ANN model and results are in log10 CFU/g

| Training Stage - TMAB | | | | | | | | |
| --- | --- | --- | --- | --- | --- | --- | --- | --- |
| EA | | | EB | | | EC | | |
| 1h | 24h | 48h | 1h | 24h | 48h | 1h | 24h | 48h |
| 1.2457 | 0.3424 | 0.2372 | 0.9411 | 0.5084 | 0.2318 | 0.5292 | 0.2232 | 0.1115 |
| 1.5713 | 0.0271 | -0.0033 | 0.7499 | 0.5392 | 0.1063 | 0.8422 | 0.3594 | 0.1893 |
| 1.5455 | 0.1468 | 0.0764 | 0.8856 | 0.4976 | 0.0778 | 0.5413 | 0.2284 | 0.1144 |
| 1.5659 | 0.0750 | 0.0287 | 1.0870 | 0.6844 | 0.2499 | 0.6258 | 0.2633 | 0.1630 |
| 1.8338 | 1.2924 | 0.7018 | 0.9411 | 0.5084 | 0.2318 | 1.6470 | 0.7117 | 0.3783 |
| 1.7908 | 1.1920 | 0.7315 | 2.2013 | 0.7337 | 0.3862 | 0.8601 | 0.3670 | 0.1902 |
| 1.8649 | 1.3188 | 0.7104 | 1.2809 | 0.9597 | 0.7365 | 0.5404 | 0.2437 | 0.2744 |
| 1.9115 | 1.2830 | 0.7442 | 2.0108 | 0.7126 | 0.3864 | 0.6570 | 0.2787 | 0.1419 |
| 1.6405 | 1.0919 | 0.7049 | 2.6709 | 1.4232 | 0.8942 | 0.2108 | 0.0773 | 0.1487 |
| 1.7610 | 1.2267 | 0.6937 | 2.2013 | 0.7337 | 0.3862 | 0.6767 | 0.2872 | 0.1466 |
| 1.8720 | 1.3220 | 0.7132 | 0.7632 | 0.5642 | 0.3572 | 1.0712 | 0.4749 | 0.3735 |
| 2.0708 | 1.4315 | 0.8380 | 1.7529 | 0.6857 | 0.3846 | 0.7448 | 0.3160 | 0.1814 |
| 2.0811 | 1.4202 | 0.7741 | 1.7229 | 0.6825 | 0.3840 | 0.5872 | 0.2483 | 0.1253 |
| 1.9441 | 1.3373 | 0.7411 | 2.1039 | 1.7006 | 1.5556 | 0.8240 | 0.3513 | 0.1871 |
| 1.3558 | 1.1912 | 0.8132 | 2.1808 | 0.7314 | 0.3863 | 0.5856 | 0.2476 | 0.1249 |
| 1.4862 | 1.0394 | 0.7071 | 1.0838 | 0.5513 | 0.2757 | 2.0081 | 0.8730 | 0.4661 |
| 1.0360 | 0.8635 | 0.6797 | 0.9867 | 0.6744 | 0.2839 | 1.0361 | 0.4437 | 0.2321 |
| Testing Stage - TMAB | | | | | | | | |
| 0.9175 | 0.8073 | 0.6621 | 1.1028 | 0.5569 | 0.2813 | 0.6345 | 0.2689 | 0.1365 |
| 1.3800 | 0.8146 | 0.5276 | 1.1879 | 0.5815 | 0.3060 | 0.7063 | 0.3002 | 0.1546 |
| 1.5085 | 1.0009 | 0.6666 | 1.0700 | 0.8570 | 0.5852 | 2.1282 | 0.9278 | 0.4958 |
| 1.6653 | 1.1058 | 0.7063 | 4.8835 | 3.3337 | 1.8724 | 1.8400 | 0.7974 | 0.4250 |
| 1.8924 | 1.3299 | 0.7209 | 1.3478 | 1.1844 | 1.0282 | 2.2642 | 1.0994 | 0.5794 |
| 1.8793 | 1.2552 | 0.7409 | 1.2592 | 0.6011 | 0.3254 | 0.6797 | 0.2885 | 0.1473 |
| 3.4277 | 2.8178 | 1.8389 | 1.4853 | 0.6016 | 0.1152 | 3.0686 | 1.4856 | 0.7232 |
| 1.2672 | 1.1201 | 0.8045 | 2.5726 | 0.7819 | 0.3846 | 1.7112 | 0.7402 | 0.3938 |

### **Table S2:** *S. platensis* extracts antimicrobial activity on TMAB modeling using ANFIS model and results are in log10 CFU/g

###

| ANFIS Training Stage - TMAB | | | | | | | | |
| --- | --- | --- | --- | --- | --- | --- | --- | --- |
| EA | | | EB | | | EC | | |
| 1h | 24h | 48h | 1h | 24h | 48h | 1h | 24h | 48h |
| 1.4813 | 0.3963 | 0.3131 | 1.1261 | 0.4735 | 0.1053 | 0.2785 | 0.0201 | 0.0134 |
| 1.4378 | 0.1076 | 0.0477 | 0.8407 | 0.5693 | 0.1299 | 0.7298 | 0.3400 | 0.0325 |
| 1.4851 | 0.0857 | 0.0533 | 0.9992 | 0.1014 | 0.0103 | 0.3028 | 0.0646 | 0.0231 |
| 1.5154 | 0.1130 | 0.0090 | 1.0798 | 0.8802 | 0.2696 | 0.2701 | 0.0502 | 0.0099 |
| 1.0257 | 0.8787 | 0.1570 | 1.1261 | 0.4735 | 0.1053 | 0.4692 | 0.2235 | 0.0446 |
| 1.8287 | 1.1905 | 0.7257 | 1.5857 | 0.7799 | 0.4667 | 0.2227 | 0.4229 | 0.2978 |
| 1.9076 | 1.3293 | 0.6530 | 1.2400 | 0.9805 | 0.7400 | 0.9001 | 0.1900 | 0.2700 |
| 1.9312 | 1.2086 | 0.5104 | 1.3476 | 0.7414 | 0.4363 | 0.9749 | 0.3355 | 0.1323 |
| 1.7100 | 1.0194 | 0.4792 | 1.5299 | 0.6700 | 0.3401 | 0.6498 | 0.2099 | 0.1902 |
| 2.0147 | 1.0198 | 0.3300 | 1.5857 | 0.7799 | 0.4667 | 1.1361 | 0.6027 | 0.2419 |
| 1.8938 | 1.3731 | 0.6644 | 0.7203 | 0.6083 | 0.4098 | 0.5000 | 0.2900 | 0.1799 |
| 2.0625 | 1.7634 | 0.6518 | 1.9896 | 0.6505 | 0.4189 | 1.2000 | 0.3500 | 0.3393 |
| 2.5711 | 1.2945 | 0.5231 | 2.1481 | 0.6438 | 0.4273 | 0.9488 | 0.2691 | 0.1417 |
| 1.8892 | 1.4038 | 0.8950 | 1.4801 | 0.7799 | 0.3699 | 0.4901 | 0.1900 | 0.2070 |
| 1.6075 | 1.3382 | 1.0010 | 1.5371 | 0.7786 | 0.4648 | 0.6366 | 0.2609 | 0.1362 |
| 1.4781 | 1.0854 | 0.6814 | 0.9581 | 0.5594 | 0.2986 | 2.2840 | 1.3106 | 0.8061 |
| 0.9533 | 0.6566 | 0.5942 | 0.9198 | 0.6794 | 0.2509 | 1.7911 | 0.1070 | 0.1002 |
| ANFIS Testing Stage - TMAB | | | | | | | | |
| 1.0199 | 0.8108 | 0.6198 | 0.9481 | 0.5706 | 0.3244 | 0.9612 | 0.4915 | 0.3920 |
| 1.9397 | 1.5516 | 0.7776 | 0.9723 | 0.6184 | 0.4367 | 1.0796 | 0.5095 | 0.2712 |
| 1.5203 | 1.2693 | 0.5906 | 1.0403 | 0.6306 | 0.5693 | 2.6870 | 0.6187 | 0.4615 |
| 1.7683 | 1.3128 | 0.4959 | 4.8802 | 3.3398 | 1.8699 | 2.4922 | 1.7637 | 0.8624 |
| 1.8668 | 1.4595 | 1.0531 | 1.3399 | 1.1798 | 1.0102 | 2.1200 | 1.3700 | 0.7100 |
| 1.9127 | 1.1935 | 1.5868 | 1.1331 | 0.6525 | 0.5211 | 1.1447 | 0.6068 | 0.3753 |
| 3.6016 | 2.7999 | 1.7996 | 0.7203 | 0.4404 | 0.3500 | 3.1000 | 1.4000 | 0.7800 |
| 1.2778 | 1.0683 | 0.9997 | 3.2419 | 0.6484 | 0.3883 | 2.3880 | 1.2910 | 0.8314 |

**Table S3:**  *S. platensis* extracts antimicrobial activity on TPAB modeling using ANN model and results in log10 CFU/g

| Training Stage - TPAB | | | | | | | | | | |  |
| --- | --- | --- | --- | --- | --- | --- | --- | --- | --- | --- | --- |
| EA | | | EB | | | | EC | | | |  |
| 1h | 24h | 48h | | 1h | 24h | 48h | | 1h | 24h | 48h | |
| 1.497 | 0.297 | 0.048 | | 1.330 | 0.920 | 0.506 | | 0.831 | 0.571 | 0.283 | |
| 1.945 | 1.176 | 0.452 | | 1.258 | 0.933 | 0.448 | | 0.760 | 0.296 | 0.066 | |
| 1.953 | 1.201 | 0.474 | | 1.246 | 0.943 | 0.435 | | 0.801 | 0.558 | 0.279 | |
| 1.949 | 1.189 | 0.463 | | 1.175 | 1.045 | 0.345 | | 0.744 | 0.255 | 0.034 | |
| 1.852 | 1.212 | 0.613 | | 1.330 | 0.920 | 0.506 | | 0.845 | 0.537 | 0.252 | |
| 2.038 | 1.418 | 0.787 | | 1.242 | 0.791 | 0.489 | | 0.685 | 0.490 | 0.245 | |
| 2.190 | 1.574 | 0.904 | | 1.624 | 1.401 | 0.541 | | 1.033 | 0.701 | 0.348 | |
| 1.644 | 1.021 | 0.521 | | 1.631 | 1.256 | 0.602 | | 0.681 | 0.484 | 0.242 | |
| 1.718 | 1.075 | 0.525 | | 1.087 | 0.775 | 0.381 | | 0.685 | 0.384 | 0.157 | |
| 2.037 | 1.386 | 0.715 | | 1.177 | 0.688 | 0.479 | | 0.642 | 0.456 | 0.228 | |
| 2.638 | 2.033 | 1.260 | | 1.022 | 0.687 | 0.366 | | 0.806 | 0.559 | 0.279 | |
| 1.292 | 1.022 | 0.842 | | 1.644 | 1.267 | 0.607 | | 0.751 | 0.520 | 0.258 | |
| 1.289 | 0.884 | 0.768 | | 1.196 | 0.718 | 0.482 | | 0.931 | 0.635 | 0.316 | |
| 1.499 | 0.875 | 0.450 | | 1.038 | 0.754 | 0.353 | | 1.744 | 1.223 | 0.612 | |
| 1.298 | 0.948 | 0.878 | | 1.061 | 0.489 | 0.467 | | 0.845 | 0.593 | 0.297 | |
| 2.389 | 1.778 | 1.062 | | 4.706 | 2.779 | 2.261 | | 3.161 | 2.252 | 1.131 | |
| 2.847 | 2.245 | 1.421 | | 4.712 | 2.89 | 2.224 | | 2.528 | 1.861 | 0.94 | |
| Testing Stage - TPAB | | | | | | | | | | |  |
| 3.37 | 2.552 | 1.299 | | 2.876 | 1.945 | 1.247 | | 3 | 2.151 | 1.081 | |
| 3.28 | 2.556 | 1.456 | | 2.23 | 1.645 | 0.892 | | 1.93 | 1.284 | 0.637 | |
| 3.305 | 2.554 | 1.412 | | 1.373 | 1.21 | 0.428 | | 2.321 | 1.587 | 0.791 | |
| 2.296 | 1.669 | 0.954 | | 1.88 | 1.443 | 0.713 | | 2.122 | 1.614 | 0.818 | |
| 3.365 | 2.588 | 1.253 | | 2.763 | 1.96 | 1.158 | | 2.607 | 1.911 | 0.964 | |
| 3.366 | 2.6 | 1.235 | | 1.57 | 1.199 | 0.578 | | 2.582 | 1.88 | 0.947 | |
| 3.372 | 2.597 | 1.243 | | 2.643 | 1.964 | 1.07 | | 1.425 | 1.009 | 0.506 | |
| 2.973 | 2.49 | 1.728 | | 1.7 | 1.313 | 0.63 | | 2.655 | 1.942 | 0.98 | |

**Table S4:** *S. platensis* extracts antimicrobial activity on TPAB modelling using ANFIS model and results are in log10 CFU/g

| ANFIS Training Stage - TPAB | | | | | | | | |
| --- | --- | --- | --- | --- | --- | --- | --- | --- |
| EA | | | EB | | | EC | | |
| 1h | 24h | 48h | 1h | 24h | 48h | 1h | 24h | 48h |
| 1.6264 | 0.2903 | 0.0001 | 0.7443 | 0.6983 | 0.1367 | 0.7914 | 0.5291 | 0.0000 |
| 1.6043 | 0.8890 | 0.0302 | 0.9600 | 0.8200 | 0.0000 | 0.7470 | 0.4212 | 0.0000 |
| 1.6028 | 0.9270 | 0.0302 | 1.3500 | 0.7800 | 0.0000 | 0.4501 | 0.1201 | 0.0000 |
| 1.6036 | 0.9080 | 0.0127 | 0.6200 | 0.4500 | 0.0000 | 0.7731 | -0.0003 | 0.0000 |
| 1.6284 | 1.1520 | 0.3349 | 0.7443 | 0.6983 | 0.1367 | 0.6712 | 0.2790 | 0.0000 |
| 1.9206 | 1.3772 | 0.8418 | 1.4044 | 0.7285 | 0.3240 | 0.8800 | 0.5100 | 0.4700 |
| 2.0914 | 1.4962 | 0.9706 | 2.1600 | 1.6800 | 0.5100 | 1.2797 | 0.7200 | 0.1900 |
| 1.3285 | 0.9649 | 0.5678 | 2.1587 | 1.4475 | 0.8717 | 0.8499 | 0.5500 | 0.2200 |
| 1.4221 | 1.0107 | 0.5596 | 1.0800 | 0.7400 | 0.5300 | 0.7000 | 0.4200 | 0.3200 |
| 1.8059 | 1.2508 | 0.6964 | 1.0435 | 0.7627 | 0.4640 | 0.7003 | 0.4850 | 0.4500 |
| 2.5468 | 1.8133 | 0.5403 | 1.2400 | 0.8900 | 0.6500 | 0.7926 | 0.5451 | 0.5100 |
| 1.2304 | 0.8294 | 0.6022 | 2.1382 | 1.4724 | 0.8962 | 0.8317 | 0.6239 | 0.3200 |
| 1.1933 | 0.8701 | 0.7424 | 1.5534 | 0.7524 | 0.4235 | 0.8229 | 0.4469 | 0.2700 |
| 1.0097 | 0.7429 | 0.5750 | 1.2800 | 1.0000 | 0.5100 | 1.1900 | 0.4100 | 0.3700 |
| 1.4257 | 1.0846 | 0.8267 | 1.0798 | 0.8378 | 0.7073 | 0.3800 | 0.2100 | 0.1200 |
| 2.2964 | 1.6389 | 1.1402 | 4.9800 | 3.6100 | 2.0800 | 3.1400 | 2.4200 | 1.1500 |
| 2.4576 | 1.9719 | 1.5384 | 5.1400 | 3.7800 | 2.0800 | 2.4500 | 2.0700 | 0.8900 |
| ANFIS Testing Stage - TPAB | | | | | | | | |
| 3.4210 | 2.8058 | 1.5176 | 2.7100 | 0.6127 | 1.2503 | 3.0400 | 1.8900 | 1.0300 |
| 2.9835 | 2.3711 | 1.3002 | 2.5769 | 1.9736 | 1.0770 | 1.9200 | 1.6900 | 0.7200 |
| 2.2416 | 2.4774 | 1.4305 | 1.2300 | 1.1000 | 0.8100 | 2.3100 | 1.3800 | 0.8200 |
| 2.1237 | 1.4912 | 0.9881 | 2.3212 | 1.6849 | 0.9119 | 2.0300 | 1.8500 | 0.7900 |
| 2.8308 | 2.0730 | 0.7885 | 2.9800 | 2.5800 | 1.8400 | 2.4100 | 2.1300 | 0.9100 |
| 2.7980 | 2.0577 | 1.3743 | 2.3245 | 1.3228 | 0.7489 | 2.1800 | 2.0700 | 0.8900 |
| 3.6700 | 2.6197 | 1.3793 | 2.2400 | 2.3200 | 1.4100 | 1.5300 | 1.4100 | 0.6100 |
| 2.7505 | 2.5270 | 1.7560 | 2.1007 | 1.5720 | 0.9119 | 2.5200 | 1.7800 | 0.9400 |

### **Table S5:** Summary of ANN model MSE and RMSE in all the simulation of *S. platensis* extracts antimicrobial activity over TMAB and TPAB modeling along the time duration

| Training | | | | | | |
| --- | --- | --- | --- | --- | --- | --- |
|  | MSE | | | RMSE | | |
|  | 1h | 24h | 48h | 1h | 24h | 48h |
| ANN-TMAB-EA | 0.1031 | 0.0506 | 0.0590 | 0.3211 | 0.2250 | 0.2428 |
| ANN-TMAB-EB | 0.3051 | 0.1291 | 0.1077 | 0.5523 | 0.3593 | 0.3282 |
| ANN-TMAB-EC | 0.2508 | 0.0373 | 0.0191 | 0.5008 | 0.1931 | 0.1382 |
| ANN-TPAB-EA | 0.1406 | 0.0706 | 0.1249 | 0.3750 | 0.2657 | 0.3534 |
| ANN-TPAB-EB | 0.1741 | 0.1669 | 0.0788 | 0.4173 | 0.4085 | 0.2807 |
| ANN-TPAB-EC | 0.0505 | 0.0841 | 0.0312 | 0.2248 | 0.2901 | 0.1767 |
| Testing | | | | | | |
| ANN-TMAB-EA | 0.0601 | 0.1373 | 0.1573 | 0.2451 | 0.3705 | 0.3967 |
| ANN-TMAB-EB | 0.1343 | 0.0185 | 0.0159 | 0.3664 | 0.1361 | 0.1260 |
| ANN-TMAB-EC | 0.3656 | 0.3033 | 0.0812 | 0.6046 | 0.5507 | 0.2850 |
| ANN-TPAB-EA | 0.4066 | 0.1372 | 0.0696 | 0.6376 | 0.3704 | 0.2638 |
| ANN-TPAB-EB | 0.2381 | 0.5373 | 0.1563 | 0.4879 | 0.7330 | 0.3953 |
| ANN-TPAB-EC | 0.0300 | 0.0753 | 0.0037 | 0.1732 | 0.2745 | 0.0609 |

### **Table S6:** Summary of ANFIS model MSE and RMSE in all the simulation of *S. platensis* extracts antimicrobial activity over TMAB and TPAB prediction along the time duration

| Training | | | | | | |
| --- | --- | --- | --- | --- | --- | --- |
|  | MSE | | | RMSE | | |
|  | 1h | 24h | 48h | 1h | 24h | 48h |
| ANFIS-TMAB-EA | 0.0400 | 0.0142 | 0.0118 | 0.2001 | 0.1191 | 0.1085 |
| ANFIS-TMAB-EB | 0.0809 | 0.0323 | 0.0026 | 0.2844 | 0.1797 | 0.0508 |
| ANFIS-TMAB-EC | 0.0143 | 0.0063 | 0.0009 | 0.1196 | 0.0795 | 0.0301 |
| ANFIS-TPAB-EA | 0.0500 | 0.0436 | 0.0304 | 0.2235 | 0.2089 | 0.1742 |
| ANFIS-TPAB-EB | 0.0570 | 0.0508 | 0.0124 | 0.2388 | 0.2255 | 0.1115 |
| ANFIS-TPAB-EC | 0.0014 | 0.0046 | 0.0000 | 0.0377 | 0.0675 | 0.0000 |
| Testing | | | | | | |
| ANFIS-TMAB-EA | 0.0144 | 0.0325 | 0.0033 | 0.1201 | 0.1803 | 0.0577 |
| ANFIS-TMAB-EB | 0.0017 | 0.0049 | 0.0011 | 0.0417 | 0.0702 | 0.0333 |
| ANFIS-TMAB-EC | 0.0283 | 0.0239 | 0.0038 | 0.1683 | 0.1547 | 0.0614 |
| ANFIS-TPAB-EA | 0.2634 | 0.0518 | 0.0228 | 0.5132 | 0.2275 | 0.1509 |
| ANFIS-TPAB-EB | 0.0182 | 0.0491 | 0.0259 | 0.1350 | 0.2217 | 0.1611 |
| ANFIS-TPAB-EC | 0.0000 | 0.0000 | 0.0000 | 0.0000 | 0.0000 | 0.0000 |

**Figures**


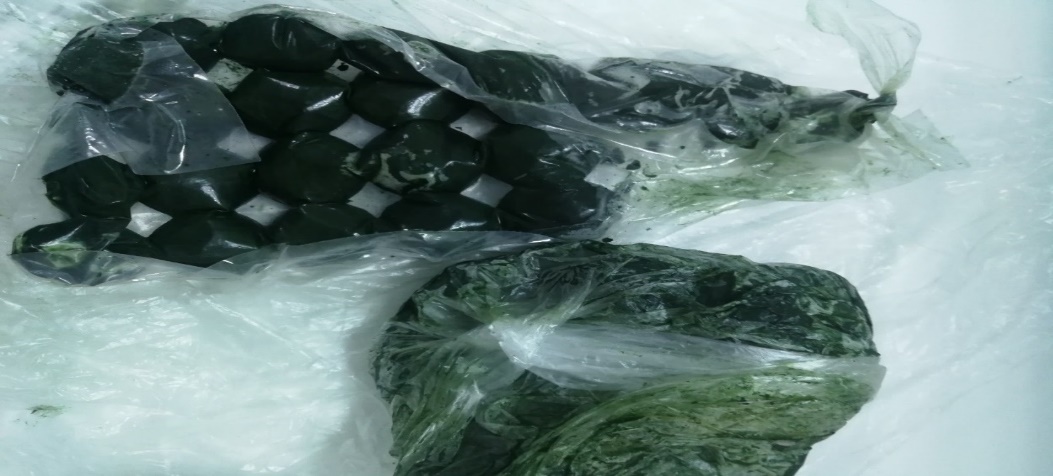


### **Fig. S1.** Fresh frozen *S. platensis* algae

- **Weighing the freeze Spirulina algae of 0.5, 1 and 5 % w/v ratio using 100 ml of distilled water as solvent and three different concentrations of EA, EB and EC were obtained**
- **The freezing and thawing temperature is -18 & 25 ^◦^C for the time interval of 2 hour and 24 hours respectively**
- **The yield contains a supernatant of extracts and residues so remove the residue by distillation and transferring in to another sterilized glass jar**
- **Hue angles of chart was used for color valuation (the extract was in the blue range (İlter et al., 2018; Kara J. et al., 2021)**

**Fig. S2.** Procedures for the extraction of *S. platensis*


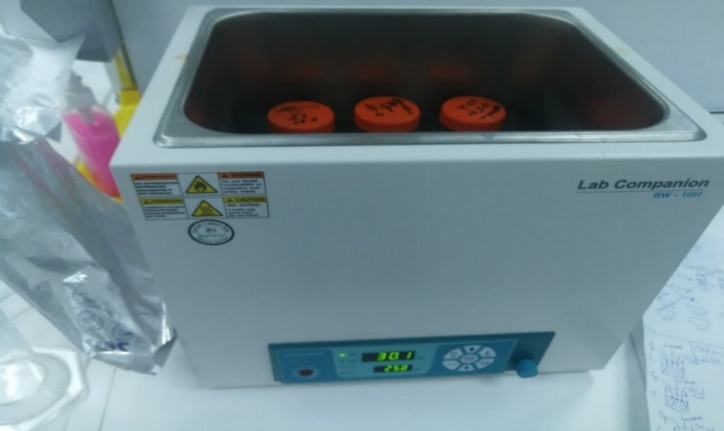


**Fig. S3.** Spirulina algae extraction using thaw method in water base at 25 ^O^C for 24 h


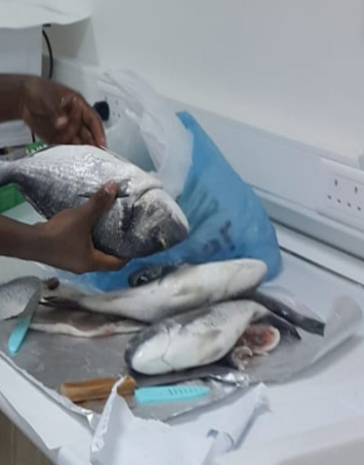

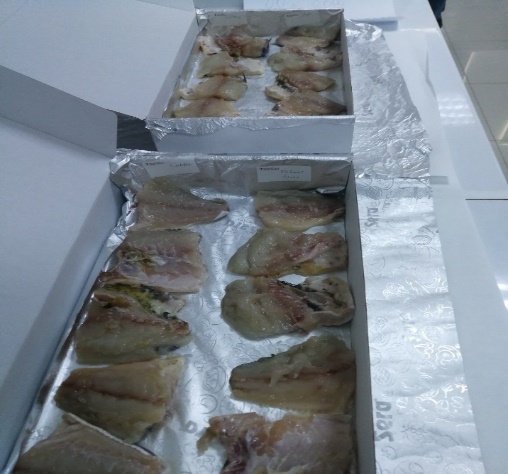


### **Fig. S4.** Fresh dead whole Nile Tilapia (*Oreochromis niloticus*) fish sample preparation

### **Fig. S5.** The regression graph of Spirulina EA antimicrobial activity over TMAB using ANN model

### **Fig. S6.** The regression graph of Spirulina EB antimicrobial activity over TMAB using ANN model

**Fig. S7.** The regression graph of Spirulina EC antimicrobial activity over TMAB using ANN model

### **Fig. S8.** Scatter plots of Spirulina EA antimicrobial activity over TMAB bacteria at different time duration using ANFIS model

### **Fig. S9.** Scatter plots of Spirulina EB antimicrobial activity over TMAB bacteria at different time duration using ANFIS model

**Fig. S10.** The regression graph of Spirulina EA antimicrobial activity over TPAB using ANN model

###

### **Fig. S11.** The regression graph of Spirulina EB antimicrobial activity over TPAB using ANN model

###

### **Fig. S12.** The regression graph of Spirulina EC antimicrobial activity over TPAB using ANN model

### **Fig. S13.** Scatter plots of Spirulina EA antimicrobial activity over TPAB at different time duration using ANFIS model

### **Fig. S14.** Scatter plots of Spirulina EB antimicrobial activity over TPAB at different time duration using ANFIS model

###

### **Fig S15.** Scatter plots of Spirulina EC antimicrobial activity over TPAB at different time duration using ANFIS model
